# Performance of an Optimized Paper-Based Test for Rapid Visual Measurement of Alanine Aminotransferase (ALT) in Fingerstick and Venipuncture Samples

**DOI:** 10.1371/journal.pone.0128118

**Published:** 2015-05-28

**Authors:** Sidhartha Jain, Radha Rajasingham, Farzad Noubary, Erin Coonahan, Ryan Schoeplein, Rachel Baden, Michael Curry, Nezam Afdhal, Shailendra Kumar, Nira R. Pollock

**Affiliations:** 1 Diagnostics For All, Cambridge, Massachusetts, United States of America; 2 Division of Infectious Diseases, Beth Israel Deaconess Medical Center, Boston, Massachusetts, United States of America; 3 The Institute for Clinical Research and Health Policy Studies, Tufts Medical Center, Boston, Massachusetts, United States of America; 4 Tufts Clinical and Translational Science Institute, Tufts University, Boston, Massachusetts, United States of America; 5 Division of Hepatology, Beth Israel Deaconess Medical Center, Boston, Massachusetts, United States of America; 6 Department of Laboratory Medicine, Boston Children’s Hospital, Boston, Massachusetts, United States of America; Emory University/Georgia Insititute of Technology, UNITED STATES

## Abstract

**Background:**

A paper-based, multiplexed, microfluidic assay has been developed to visually measure alanine aminotransferase (ALT) in a fingerstick sample, generating rapid, semi-quantitative results. Prior studies indicated a need for improved accuracy; the device was subsequently optimized using an FDA-approved automated platform (Abaxis Piccolo Xpress) as a comparator. Here, we evaluated the performance of the optimized paper test for measurement of ALT in fingerstick blood and serum, as compared to Abaxis and Roche/Hitachi platforms. To evaluate feasibility of remote results interpretation, we also compared reading cell phone camera images of completed tests to reading the device in real time.

**Methods:**

96 ambulatory patients with varied baseline ALT concentration underwent fingerstick testing using the paper device; cell phone images of completed devices were taken and texted to a blinded off-site reader. Venipuncture serum was obtained from 93/96 participants for routine clinical testing (Roche/Hitachi); subsequently, 88/93 serum samples were captured and applied to paper and Abaxis platforms. Paper test and reference standard results were compared by Bland-Altman analysis.

**Findings:**

For serum, there was excellent agreement between paper test and Abaxis results, with negligible bias (+4.5 U/L). Abaxis results were systematically 8.6% lower than Roche/Hitachi results. ALT values in fingerstick samples tested on paper were systematically lower than values in paired serum tested on paper (bias -23.6 U/L) or Abaxis (bias -18.4 U/L); a correction factor was developed for the paper device to match fingerstick blood to serum. Visual reads of cell phone images closely matched reads made in real time (bias +5.5 U/L).

**Conclusions:**

The paper ALT test is highly accurate for serum testing, matching the reference method against which it was optimized better than the reference methods matched each other. A systematic difference exists between ALT values in fingerstick and paired serum samples, and can be addressed by application of a correction factor to fingerstick values. Remote reading of this device is feasible.

## Introduction

Point-of-care (POC) diagnostics are particularly desirable for diagnosis and medical management in resource-constrained settings. In such settings, the centralized laboratory testing upon which clinicians in resource-rich settings rely may be unavailable or cost-prohibitive. If centralized testing is available, lengthy results turn-around time can lead to patients becoming lost to follow-up, adversely impacting outcomes. With these barriers in mind, there has been a recent explosion in the development of POC diagnostics for many applications [1–3].

One example of a defined need for POC testing is for monitoring of transaminases for diagnosis and management of drug-induced liver injury (DILI). Transaminase testing is particularly necessary for persons on medications for treatment of HIV and TB, as many of these medications are known to be hepatotoxic [4,5]. Transaminase monitoring is also valuable to evaluate those with underlying liver diseases such as hepatitis B or hepatitis C. Collectively, these diseases disproportionately affect those in resource-limited settings, and with limited or no access to transaminase testing in many of these settings, patients are put at increased risk of complications of DILI. Typically, transaminase monitoring requires equipment for a venous blood draw, a trained phlebotomist, centrifugation to separate serum or plasma, and testing on a large automated platform. Such platforms are expensive and require highly trained technicians for testing and maintenance, making them impractical for use and scale-up in many developing countries. Because of these obstacles, in many resource-limited settings, patients on potentially hepatotoxic medications receive minimal or no monitoring during treatment. Automated platforms for POC transaminase testing have been developed (Roche Reflotron and Alere Cholestech); however, the Choletech ALT test is currently off the market worldwide and the Reflotron is currently off the market in the US.

We have recently developed a paper-based, multiplexed, microfluidic assay to visually and semi-quantitatively measure alanine aminotransferase (ALT) in a fingerstick sample [6–8]. This device is based on “patterned-paper” technology, in which a wax-based printer is used to create a series of hydrophobic barriers and hydrophilic channels that guide fluid wicking through the paper both laterally and vertically. As fluid flows directionally through the layers of patterned paper, it contacts zone-specific assay reagents, allowing multiple reactions to be performed in parallel on a single sample. Our assay has been designed to yield a rapid (~18–30’), semi-quantitative, visual result; moreover, it is portable, disposable, requires no power, and is anticipated to cost ~$0.10/test, making it ideal for resource-limited settings. Similar paper-based microfluidic technology is being evaluated for a wide range of applications, including (as examples) glucose and protein measurement, detection of bacteria, detection of hepatitis C antibody, detection of cancer biomarkers, and blood typing [9–15]. To our knowledge, within this emerging class of paper-based microfluidic platforms, our paper-based ALT test is currently the closest to actual clinical application.

Early pre-clinical testing on clinical serum and whole blood specimens demonstrated that the paper-based device could yield visual measurements with >90% accuracy [6] and was therefore ready for field testing. Thereafter, the first fingerstick evaluation of the test was performed in 600 HIV-infected persons receiving care in a busy HIV clinic in Vietnam [7], considered an ideal target setting for application of this test. That evaluation study demonstrated that the device operation and reading process were both feasible and extremely reproducible in this target setting, but highlighted the need for further device optimization to reduce hemolysis rates and improve accuracy. The device subsequently underwent extensive optimization, including sourcing of a new plasma separation membrane, treatment of this membrane with an anti-hemolytic coating to reduce hemolysis rates, reformulation of assay chemistry, and recalibration against an automated reference standard, Abaxis Piccolo [16].

Here, we present results of a validation study of the optimized ALT test as performed in ambulatory outpatients with liver disease or on hepatotoxic medications, each of whom required ALT monitoring as part of routine care. The goals of this study were to evaluate the performance of the optimized paper-based test for measurement of ALT levels in both fingerstick blood and venipuncture serum for patients with a range of baseline ALT levels, as compared to results of testing the serum on two FDA-approved automated platforms in wide clinical use. As a result, we were able to evaluate the impact of sample type on results of the paper test, as well as to compare performance of the paper test to that of each automated platform. We also assessed the potential utility of cell-phone cameras to allow remote interpretation of the paper test results.

## Materials and Methods

### Device Design, Production and Storage

**Fig 1:**
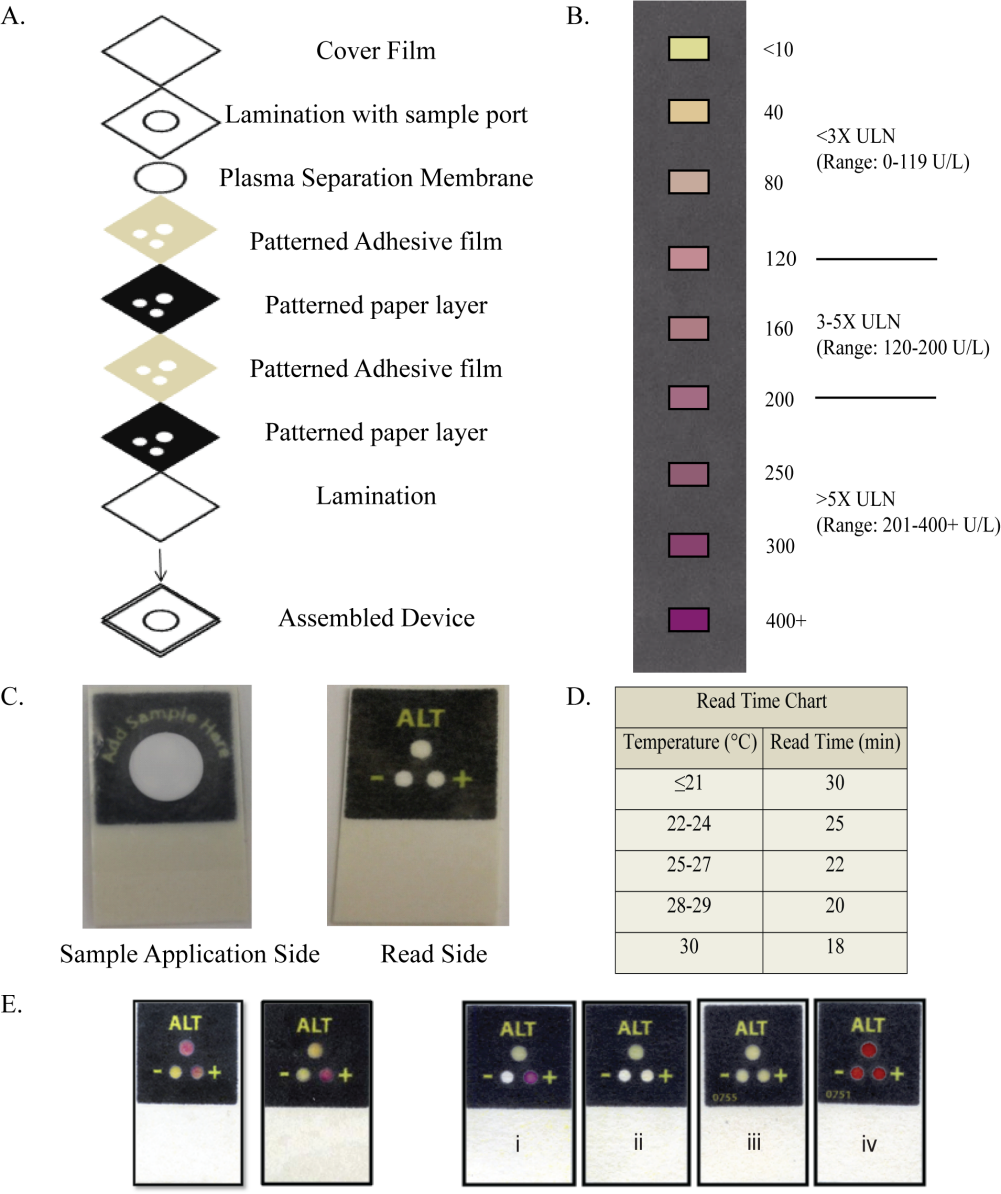
Schematic of the 3-Zone DFA paper-based alanine aminotransferase (ALT) test. A. The DFA ALT test is constructed by assembling two patterned paper layers, a plasma separation membrane disc, and protective lamination films to create a 3-dimensional device. A cover film is applied to devices to be used with fingerstick whole blood samples; this cover film is not applied to devices to be used with serum samples. All layers are adhered together using patterned, pressure-sensitive adhesive films. B. The test utilizes a peroxidase-based colorimetric assay to provide a semi-quantitative ALT result determined through visual comparison with a reference color chart. The color chart also allows the user to place the ALT result in one of three categorical bins: <3X upper limit of normal (ULN), 3-5X ULN, and >5X ULN. C. A fingerstick blood sample is applied to the sample port on the “sample application side” of the test. Blood cells are separated by the plasma separation membrane, allowing plasma to wick into the device and react with reagents dried onto individual detection zones; results are viewed on the “read side.” D. The results are read after an incubation time that corresponds to the ambient temperature. E. Two control zones on the test are used to determine the validity of the test results. Examples of valid devices are shown in the two images on the left. Four invalid examples are shown on the right, as follows: i. Insufficient sample volume, evident by the lack of a yellow color in the negative control zone. ii. Insufficient sample volume (as in i) and positive control failure, latter indicating inactive reagents at the time of testing. iii. Positive control failure. iv. Hemolyzed sample, indicated by the presence of a red color in the negative control zone.

The “patterned paper” ALT tests are created using wax-based printing technology; after printing, paper layers are heated to 110°C, which melts the wax and allows it to permeate through the thickness of the paper (GE Whatman Chromatography Grade 1, Piscataway, NJ), creating microfluidic, hydrophilic detection zones surrounded by hydrophobic wax barriers [17–20]. The ALT test is constructed by stacking two such patterned paper layers, along with a plasma separation membrane disc (Primecare NX-membrane, International Point of Care Inc. Toronto, CA) and lamination films, to create a 3D device (Fig 1A). A cover film (2MIL low density polyethylene, Warp Bros, Chicago IL) providing protection against sample evaporation is added to devices to be used with fingerstick whole blood samples; this cover film is not applied to devices to be tested using serum. The layers are adhered together using patterned pressure-sensitive adhesive films (Flexcon Inc, Spencer, MA). The plasma separation membrane separates red and white blood cells from plasma within fingerstick whole blood, allowing plasma to wick to the detection zones. The plasma separation membrane is treated with an anti-hemolytic coating to prevent hemolysis of cells during filtration.

The ALT test utilizes a peroxidase-based colorimetric assay [6], producing a red color in the presence of elevated ALT. The intensity of the red color of the test spot is directly proportional to the concentration of ALT in the sample, allowing semi-quantitative, visual interpretation of the results by comparison with a reference color chart or “read guide” (Fig 1B). The read guide also allows the user to place the ALT result in one of three clinically relevant bins [<3X upper limit of normal (ULN), 3-5X ULN, and >5X ULN] used in the management of patients at risk for DILI. The negative control spot serves as an indicator for sample adequacy (adequate sample volume, no hemolysis), and the positive control spot serves as an indicator for reagent activity at the time of testing (Fig 1C and 1E). In this assay, as in any other enzymatic reaction, the rate of the reaction is temperature-dependent. Therefore, the time window within which the test must be read is determined using a temperature/read-time chart (Fig 1D). Each control zone is interpreted as either ‘VALID’ or ‘INVALID’. An ‘INVALID’ on either control zone invalidates the device [7] (Fig 1E).

400 devices were fabricated in parallel at Diagnostics For All (DFA) using established techniques [6,7]. The paper test measures ALT via its enzymatic activity in a three-step reaction. The test zone contains L-alanine and α-ketoglutarate in the first layer (as substrates) and ALT, present in the sample, catalyzes the transfer of an amino group from L-alanine to α-ketoglutarate producing pyruvate and glutamate. In the second step, pyruvate oxidase, contained in the second layer of the ALT test zone, oxidizes the pyruvate to acetylphosphate and hydrogen peroxide. In the final step of the reaction, horseradish peroxidase (HRP) catalyzes the reaction of the hydrogen peroxide with 4-aminoantipyrine and m-tolyldiethanolamine (TDA) to produce a red/pink colored dye complex that correlates to the ALT concentration in the sample. This enzymatic reaction is similar to that used in other ALT tests, specifically Cholestech LDX (Alere, Inc.), and Reflotron System (Boehringer Mannheim Corp.).

The chemical reactions are as follows:

$$\text{L-alanine}+\mathrm{\alpha -}\text{ketoglutarate}\overset{\text{Alanine Aminotransferase}}{\to }\text{pyruvate}+\text{glutamate}$$

$$\text{pyruvate}+\text{phosphate}+{\text{O}}_{2}+{\text{H}}_{2}\text{O}\overset{\begin{array}{c} \text{Pyruvate Oxidase}\\ \text{TPP},\;\text{Mg}2+ \end{array}}{\to }\text{acetylphosphate}+{\text{CO}}_{2}+{\text{H}}_{2}{\text{O}}_{2}$$

$${\text{H}}_{2}{\text{O}}_{2}+4\text{-aminoantipyrine}+\text{m-tolyldiethanolamine}\overset{\text{HRP}}{\to }\text{Red}/\text{Pink Dye Complex}$$

This formulation produces a stronger color change and larger linear dynamic range when compared to the formulation relying on 4-aminoantipyrine and 3,5-diaminobenzoic acid (DABA) used in previous versions of the DFA paper ALT test [6–8].

In the optimized paper test, spotting of alanine and α-ketoglutarate in a separate layer from the remaining reagents improved stability and performance of the paper test. A stabilizer (bovine serum albumin) was added to all reagents to extend shelf life. Accelerated stability studies showed that the device is stable for approximately 18 months when stored at the recommended temperature range (18–30°C).

The devices were pouched individually in foil-lined aluminum pouches, each with a pillow pack of desiccant, and stored at room temperature for the duration of the study. Devices were quality-tested prior to release for use in this study, per DFA’s standard operating procedures.

### Within-run precision study

Three standards were prepared by spiking normal human serum (Valley Biomedical Inc, Winchester, VA) with purified ALT (LeeBio Solutions, St. Louis, MO) at target concentrations (low, medium and high). These samples were blinded to the operator running the precision studies. The three standards were each tested in five replicates on the Hepatic Function Panel, Abaxis Piccolo Xpress (Abaxis, Union City, CA)) [16] and in 10 replicates on the DFA paper tests on the same day. The % coefficient of variation (CV) was calculated for both Abaxis Piccolo and DFA paper tests. This precision study was performed on one lot each of Abaxis Piccolo Hepatic Function Panels and DFA paper devices.

### Capillary tube dispensation volume accuracy

A 35μL Microsafe capillary tube (Safe-Tec Inc, Ivyland, PA) was used to collect and dispense fingerstick samples in the clinical study. The dispense volume accuracy of the capillary tube was tested using freshly collected whole blood (Research Blood Components LLC, Brighton, MA), and de-ionized water. The weight of the dispensed volume was measured using a calibrated, analytical balance (NewClassic MF, Mettler Toledo, Columbus OH). %CV was calculated for both de-ionized water and whole blood.

### Ethics Statement

This study was approved by the Beth Israel Deaconess Medical Center (BIDMC) Institutional Review Board. The IRB approved use of verbal (rather than written) consent for this minimal risk study; a verbal consent script describing study purpose and procedures was read to each individual, verbal consent was obtained and documented, and the subject was given a study information sheet to keep.

### Participants

Participants were recruited from the BIDMC Liver Center and Infectious Diseases outpatient clinics. To be considered for study participation, participants had to be receiving ALT testing that day per clinical routine (collection of venipuncture blood, followed by testing on Roche/Hitachi platform). Recruitment was based on previous ALT results, in order to include patients with ALT values throughout the clinical range; patients with prior ALT ≥120 U/L were prioritized for recruitment when possible. Participants were sampled consecutively based on the above selection criteria, and verbal informed consent was obtained. Data on each participant were collected via chart review, and included basic demographic information, results of recent laboratory testing (including ALT levels on the day of enrollment) and primary cause of liver disease if known.

### Testing procedure

Fingersticks were performed on study participants by operator 1 using a Surgilance SLN300 lancet (Medipurpose, Duluth, GA). For the first 42 patients, the first drop of fingerstick blood was collected [using a 35μL Microsafe capillary tube (Safe-Tec Inc, Ivyland PA)] and dispensed on the device. For the next 54 patients, the first drop of blood was wiped away and the 35μL sample was collected from the second drop of blood using the capillary tube and dispensed on the device. A count up timer was started after the application of sample. After the fingerstick, patients were sent to the phlebotomy laboratory for venipuncture for routine clinical ALT testing. Operator 1 performing the fingerstick recorded the degree of difficulty (if any) in obtaining the required sample volume and the temperature/relative humidity of the fingerstick room on a study form specific to the study ID number. The device was placed in a petri dish and brought (along with timer and study form) to the incubation room where a second operator (blinded to any prior patient ALT values) interpreted and recorded the results. Operator 2 selected a read time based on the temperature of the incubation room. Results were reported in U/L (rounded to the nearest 10 U/L). Operator 2 also noted the validity of the device by visually inspecting the control zones for hemolysis, incomplete filling, and reagent function. Incubation room temperature and relative humidity were also recorded.

Following reading of device results, operator 2 photographed the device and read guide together to allow remote reading of the test images. The photographs were taken using two cell phones, one with an 8 mega pixel camera (Samsung Galaxy SII) and another with a 2 mega pixel camera (AT&T Z431). Both sets of camera phone images were texted to two separate email addresses. Two separate operators (operators 3 and 4) read the two image sets using the read guide captured in the images (operator 3 read images from the 2 mega pixel camera and operator 4 read images from the 8 mega pixel camera) on standard laptop monitors. Both operators 3 and 4 were blinded to the results noted by operator 2 in real time.

ALT testing of venipuncture blood was performed by the BIDMC clinical laboratory (serum, Roche/Hitachi platform, without pyridoxal phosphate activation [21]) as per clinical routine. The clinical serum sample was held at 4°C for 5 days and then de-identified and provided to DFA for testing on the DFA paper device and the Abaxis Piccolo analyzer [16]. Testing on the DFA paper devices was performed by another trained reader blinded to both the Piccolo results and to all other results collected in the study.

### Statistical Analysis

Agreement between methods of measurements was assessed graphically and through the estimation of the mean difference, or bias, and 95% limits of agreement. Graphs included “diagonal plots” (where one method was simply plotted against the other and evaluated relative to the line of equality) and Bland-Altman plots [22]. For Bland-Altman analysis, logarithmic transformation was used for comparisons in which the mean and standard deviation of the differences were not constant throughout the range of measurement; results were then back-transformed to give percentage differences.

For diagonal plots, all data points were included. Because the DFA paper test is not reliably linear above 250 U/L for fingerstick blood samples, for all Bland-Altman analyses for platform comparisons which included the DFA paper test, pairs of data points in which one of the values was >250 U/L were excluded from analysis, so as not to inappropriately distort the analysis of difference. Data for calculation of the correction factor was similarly truncated. Data for comparison of real-time vs remote results reading was not truncated in this manner.

A correction factor (calibration equation) was fitted using linear regression to predict the paper test result for a serum sample from a paper test result for a fingerstick sample (linear regression was used because the outcome, serum ALT concentration as measured by the DFA paper test, was treated as a continuous variable.)

## Results

### Study Setting and Participants

96 participants were recruited from the BIDMC Liver Center and the BIDMC Infectious Diseases clinic between February and June 2014. The median age of participants was 56 (range 22 to 79), and 68% were male. The median clinical ALT value (Roche/Hitachi) was 94 U/L (range 18 to 752 U/L). Sixty participants had hepatitis C virus infection, 8 had HIV infection, and 2 had hepatitis B virus infection. Other etiologies of liver disease in this population included autoimmune hepatitis (n = 2), hemochromatosis (n = 2), non-alcoholic fatty liver disease (n = 3), alpha-1 antitrypsin deficiency (n = 2), and acute liver failure secondary to medication (n = 1).

### Study testing

The median temperature in the room where the fingerstick was performed was 22.7°C (range 19.5°C to 25.9°C), with median humidity of 26% (range 15% to 47%). Median temperature in the room where the device was read was 23°C (range 22°C to 26°C), with median humidity of 21% (range 15–47%). The median time that elapsed before devices were transported from the room where the fingerstick was performed to the room where the device was read was 2 minutes and 30 seconds. All devices were incubated for the appropriate total length of time per the temperature/read-time chart (Fig 1D); incubation time was based on temperature in the reading room (Methods), and median incubation time was 25 min (range 22 to 25 min).

Most patients proceeded directly to the clinical laboratory for venipuncture immediately after providing a fingerstick sample. A small number had their venipuncture done immediately prior to fingerstick, or while waiting for their clinic visit due to delays in clinic schedules (for these latter patients, venipuncture was performed within 2 hours of fingerstick). For all patients, the venipuncture sample (serum) was tested on the Roche/Hitachi platform (per BIDMC clinical routine) on the same day that it was drawn. The serum was subsequently captured for research testing with both the DFA paper test and the Abaxis Piccolo automated platform (Methods); the serum was stored for a median time of 5 days (almost all samples were tested within 7 days) prior to this additional testing.

Three out of the 96 participants were initially enrolled but ultimately did not have ALT testing performed on venipuncture blood; two forgot to get labs done, and one was unable to get labs done due to difficulty with venipuncture. Fingerstick samples from three individuals generated invalid results on the paper test due to filling failure in the negative control zone (Fig 1E). No invalid tests due to hemolysis or positive control failure were observed. Ultimately, 91 patients provided both a valid fingerstick sample and a venipuncture serum specimen for ALT testing (Roche/Hitachi). Discarded serum was available for capture on 88 of these 91 patients (for testing on the DFA paper test and the Abaxis Piccolo). No adverse events were reported.

### Comparative performance of paper and automated tests for measurement of ALT

Prior to our clinical evaluation, a within-run precision study was performed on both the Abaxis Piccolo analyzer and the DFA paper test; results of this evaluation are shown in Table 1. The data indicate that the Abaxis Piccolo and the DFA paper test are comparable in performance and generate reproducible results. The slightly higher %CVs reported for the paper test (vs Abaxis) are expected as the paper test is a colorimetric, semi-quantitative test, whereas the Abaxis Piccolo assay is a quantitative test. \

**Table 1:** Precision of the DFA paper-based ALT test and the Abaxis Piccolo ALT test, as performed on serum standards.

	Serum Standard 1	Serum Standard 2	Serum Standard 3
**ALT (U/L) Abaxis Piccolo, mean ± SD (%CV)**	61.8 ± 3.77 (6.10)	152.4 ± 2.51 (1.65)	264.8 ± 5.26 (1.99)
**ALT (U/L) DFA paper, mean ± SD (%CV)**	60 ± 10.54 (17.57)	141 ± 9.94 (7.05)	254 ± 8.43 (3.32)

(DFA, Diagnostics For All; ALT, alanine aminotransferase; SD, standard deviation; CV, coefficient of variation)

As noted (Methods), for the first 42 patients, the first drop of fingerstick blood was collected and applied to the DFA paper test. Because initial results already suggested that fingerstick results were systematically lower than paired serum results (below) and because some (but not all) fingerstick testing protocols suggest wiping away the first drop of blood to avoid collection of excess tissue fluid, we adjusted our procedure for the next 54 patients so that the first drop of blood was wiped away and the second of drop of blood was collected and applied to the test. To evaluate whether this change in fingerstick procedure had any impact on our results, a two-sample t-test was performed to compare the average differences between DFA paper (fingerstick) and Abaxis Piccolo (serum) test results in the groups undergoing the two different procedures. The p-value for that comparison was 0.65, indicating that the average difference was unaffected by the change in the sample collection procedure; therefore, results from the two procedures were pooled for analysis.

**Fig 2:**
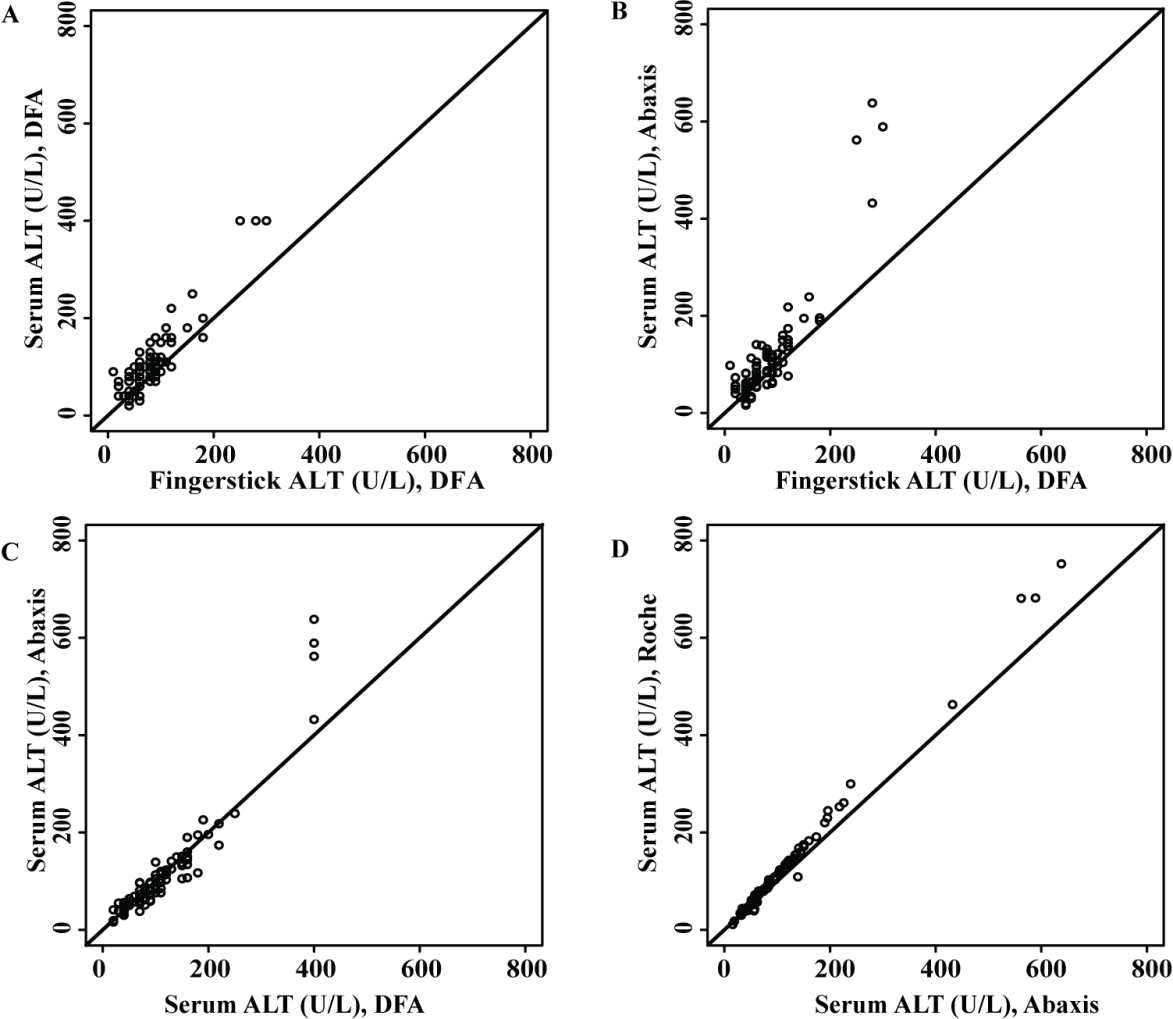
Plots of ALT results generated by different platforms for fingerstick or serum samples. The diagonal black line represents the line of equality. A. Comparison of DFA paper test results for fingerstick blood vs paired serum. B. Comparison of DFA paper test results for fingerstick blood to Abaxis Piccolo test results for paired serum. C. Comparison of DFA paper test results for serum to Abaxis Piccolo test results for serum. D. Comparison of the results of two automated platforms (Abaxis Piccolo and Roche/Hitachi) for serum samples.

**Fig 3:**
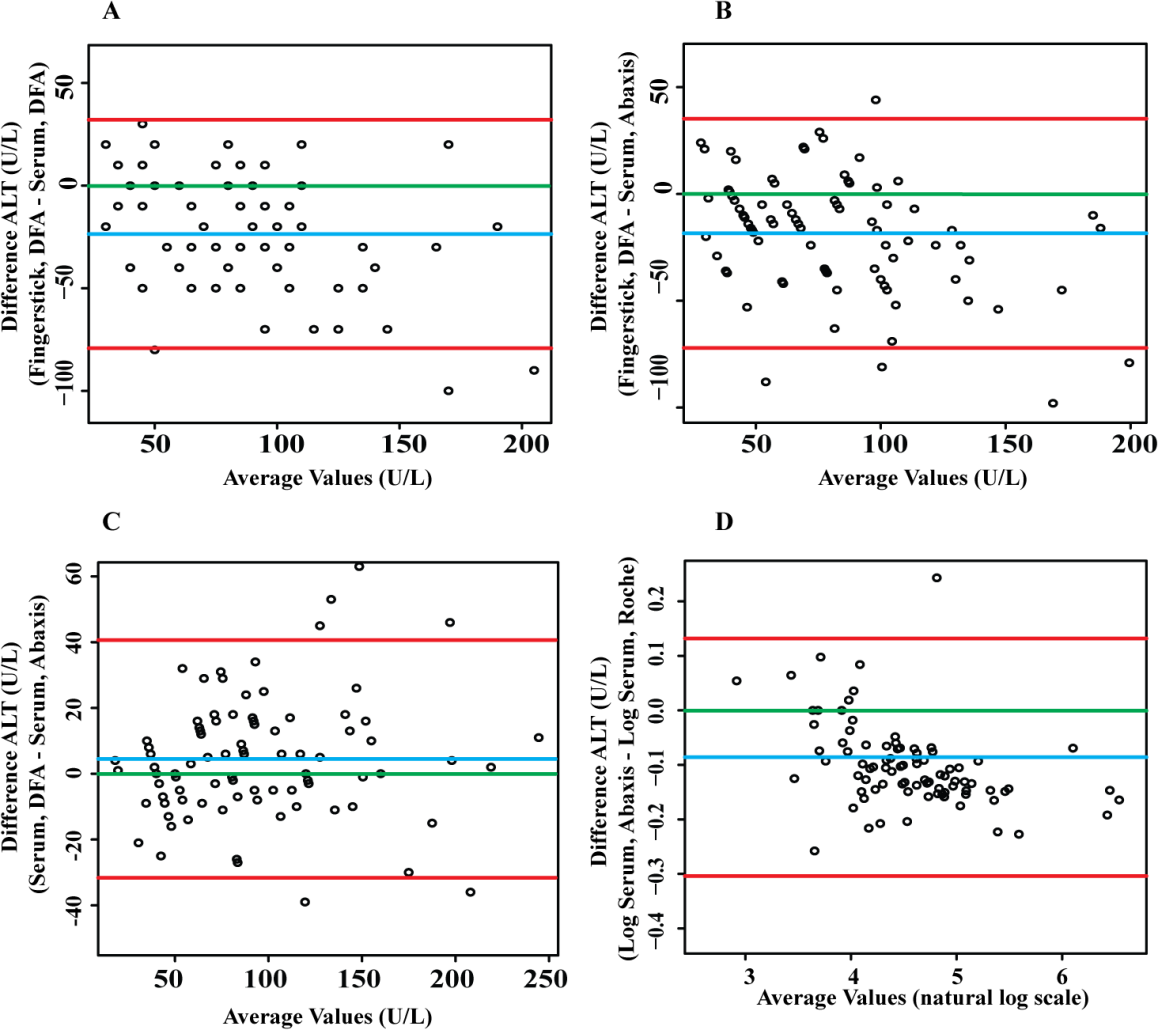
Bland-Altman plots to evaluate agreement between ALT results generated by different platforms for fingerstick or serum samples. The blue line in each plot represents the “bias” or average difference between the two methods. The red lines represent the 95% limits of agreement. The green line represents the line of no bias. A. Difference between DFA paper test results for fingerstick blood vs paired serum. B. Difference between DFA paper test results for fingerstick blood and Abaxis Piccolo test results for paired serum. C. Difference between DFA paper test results for serum and Abaxis Piccolo test results for serum. D. Difference between the log transformation of the results of the two automated platforms (Abaxis Piccolo and Roche/Hitachi) for serum samples.

ALT results from testing fingerstick and paired serum samples on the DFA paper test were compared to each other and to results from testing serum on the two automated platforms (Figs 2 and 3). The plots in Fig 2 show results for direct comparison of ALT results from two different tests performed on samples from one individual: fingerstick blood on the DFA paper test vs serum on the DFA paper test (Fig 2A), fingerstick blood on the DFA paper test vs serum on the Abaxis Piccolo (Fig 2B), serum on the DFA paper test vs serum on the Abaxis Piccolo (Fig 2C), and serum on the Abaxis Piccolo vs serum on the Roche/Hitachi platform (Fig 2D). Fig 2A shows that ALT results for fingerstick blood tested on the DFA paper test were systematically lower than ALT results for paired serum samples tested on the DFA paper test, as the majority of the data points lie above the line of equality. Similarly, ALT results for fingerstick samples were systematically lower than results for paired serum samples tested on the Abaxis Piccolo (Fig 2B), the automated platform against which the DFA paper test was calibrated during optimization. In order to ensure that the optimal fingerstick sample volume was being reproducibly delivered to the test by the plastic capillary tube used for sample collection (Methods), we evaluated the precision and accuracy of the capillary tube for dispensation of expected volumes of water and blood (Methods). The mean volume dispensed for water samples was 35.6 μL with a %CV of 2.79% (n = 100), and the mean volume dispensed for blood samples was 33.9 μL with a %CV of 2.97% (n = 100), indicating that volumes of fingerstick blood dispensed on the devices during the study were reliable.

For serum samples, ALT results from the DFA paper test showed excellent agreement with results from the Abaxis Piccolo analyzer (Fig 2C). Interestingly, ALT results from the Abaxis Piccolo were systematically lower than ALT results from the same sample tested on the Roche/Hitachi platform (Fig 2D).

Next, we evaluated agreement (for the same comparisons) by Bland-Altman analysis [22] (Methods; Fig 3). As noted, ALT values in fingerstick samples tested on the DFA paper test were systematically lower than values in paired serum tested on the DFA paper test (bias -23.6 U/L, Fig 3A, Table 2) or Abaxis (bias -18.4 U/L, Fig 3B, Table 2). For serum, there was excellent agreement between paper test and Abaxis results, with negligible bias (+4.5 U/L, Fig 3C, Table 2). Abaxis results for serum were 8.6% lower than Roche/Hitachi results on average (Fig 3D, Table 2); notably, a similar systematic difference between these platforms has also been demonstrated in proficiency testing performed by labs using these platforms (www.api-pt.com; see discussion). Logarithmic transformation was only required for the Abaxis versus Roche/Hitachi comparison (Fig 3D; see Methods). To facilitate a direct comparison of the relative bias values for serum on the DFA paper test versus Abaxis and serum on the Abaxis versus Roche/Hitachi, we also calculated the mean bias on the logarithmic scale for serum on the DFA paper test versus Abaxis and back-transformed to give percentage differences (Table 2). Serum on the DFA paper test was 2.4% higher on average than Abaxis, but this was not significantly different from no bias (95% CI for bias: -2.4% to 7.6%), while there was a significant difference between Abaxis and Roche/Hitachi (mean bias -8.6%, 95% CI -10.7% to -6.5%) (Table 2).

**Table 2:** Bias values and associated 95% confidence intervals (CI) for Bland-Altman comparisons shown in Fig 3A–3D.

	Mean bias value, U/L	95% CI	Mean bias value, %	95% CI
**A. Fingerstick, DFA—Serum, DFA**	-23.6	-29.7 to -17.4	n/a	n/a
**B. Fingerstick, DFA—Serum, Abaxis**	-18.4	-24.3 to -12.5	n/a	n/a
**C. Serum, DFA—Serum, Abaxis**	4.5	0.6 to 8.5	2.4	-2.4 to 7.6
**D. Log Serum, Abaxis—Log Serum, Roche**	n/a	n/a	-8.6	-10.7 to -6.5

n/a, not applicable.

Given the systematic difference observed between ALT concentration ([ALT]) in fingerstick vs serum samples from the same individual, a correction factor regression equation was calculated for the DFA paper test for future use to match fingerstick blood to serum ALT results, as follows:

Serumpaper[ALT]=14.81+1.12*Fingerstickpaper[ALT]

P-values for the regression coefficients were 0.03 (for the intercept, i.e. 14.81) and <0.01 (for the fingerstick paper [ALT], i.e. 1.12). We also assessed whether inclusion of a quadratic term would contribute significantly to the fit of the model by conducting a likelihood ratio test, but, as it did not, our final model contains only a linear term for the fingerstick on paper ALT value.

### Remote results interpretation

**Fig 4:**
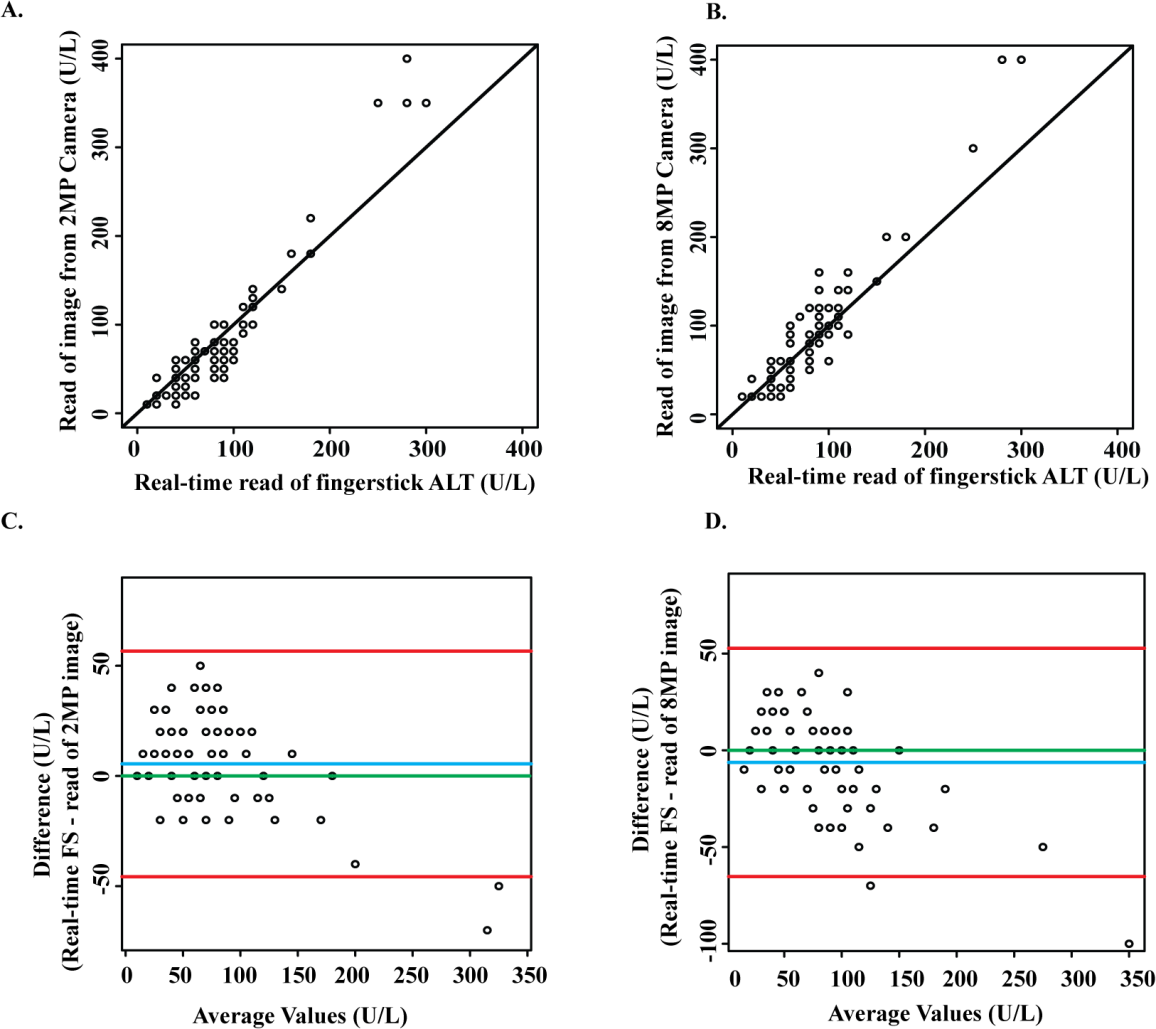
Remote vs real-time reading of DFA paper ALT test results (for fingerstick samples). A. Plot of real-time reads vs reads of 2 MP images. B. Plot of real-time reads vs reads of 8 MP images. For both A and B, the diagonal solid line represents the line of equality. C. Bland-Altman plot of differences between real-time reads and reads of 2 MP images. D. Bland-Altman plot of differences between real-time reads and reads of 8 MP images. For both C and D, the blue line represents the bias/average difference between results obtained by the two reading methods. The red lines represent the 95% limits of agreement. The green line represents the line of no bias.

We compared device results as read in real time to results read from images of resulted devices/read guides captured by cell phone cameras of different resolution (2MP vs 8MP, selected as representative of cell phone cameras commonly used in the developing and the developed world, respectively) and texted to an off-site reader (Methods). To evaluate agreement, data were plotted directly (Fig 4A and 4B) and differences were evaluated by Bland-Altman analysis (Fig 4C and 4D). There was excellent agreement between the read made in real time and the read of either the 2MP camera phone image (bias +5.5 U/L, 95% CI 0.1 to 10.9) or the 8MP camera phone image (bias -6.2 U/L, 95% CI -12.4 to -0.0) (Fig 4C and 4D).

## Discussion

The results of our study indicate that the performance of the DFA paper ALT test is comparable to that of standard automated platforms widely used in clinical labs around the world. We found that the DFA paper test was highly accurate for serum testing, matching the reference method against which it was optimized (Abaxis) better than the two reference methods (Abaxis and Roche/Hitachi) matched each other. The systematic ~9% difference we observed between serum ALT results measured by the Abaxis Piccolo vs Roche/Hitachi platforms is very similar to the difference observed on standardized proficiency testing performed by working clinical laboratories utilizing these two platforms (www.api-pt.com). This observation should serve as a reminder to clinicians that, in the absence of international standards for ALT measurement, any given automated platform does not necessarily provide the “right answer.” We acknowledge that serum was tested on the Abaxis platform after it was tested on the Roche/ Hitachi platform (typically ~5 days later), but also note that ALT values measured in serum stored at 4°C remain stable for approximately one week [23].

Importantly, we noted a systematic difference between ALT values measured in fingerstick vs paired serum samples, with measured ALT values in fingerstick blood consistently below measured ALT values in paired serum. This difference suggests an inherent difference in ALT values between capillary and venous blood, as has been observed for several other analytes, e.g. [24–27]. Our findings may be specific to the DFA paper ALT test, as existing FDA-approved POC platforms for measurement of ALT in fingerstick blood (Roche Reflotron [28] and Alere Cholestech LDX [29,30]) report no systematic bias or difference between capillary and venous blood. However, review of available information summarizing the performance of these two tests suggests that these tests were not actually validated with fingerstick samples from patients with elevated ALT values, but rather only with fingerstick samples from patients with normal to mildly elevated (e.g. maximum of 65 U/L) ALT values [28–30]. The systematic difference we observed between fingerstick blood and serum could easily have been missed if testing had only included patients with normal to mildly elevated ALT values. Given the absence of data comparing ALT levels in fingerstick vs serum samples in patients with elevated ALT, we had no apriori expectation of how well paper-based test results for these two sample types would agree. To our knowledge, this is the first study to evaluate the performance of a POC transaminase test on both fingerstick and serum samples in a patient population with a wide range of baseline ALT values. Unfortunately, our study was limited by the fact that the Alere Cholestech LDX was recalled by the FDA just prior to the start of this study, thus preventing the direct comparison between ALT results measured in fingerstick samples on the two platforms. Evaluation of the limited additional literature on these platforms is complicated by the use of different sample types, sample collection methods, and comparator methods. In an independent clinical trial report [31], Green et al compared serum ALT values measured by a Roche-Hitachi Modular Analyzer and paired whole blood ALT values measured by Cholestech LDX, and found that ALT values measured by Cholestech were systematically lower than those measured by Roche/Hitachi. However, another study by James et al [32] noted higher whole blood ALT values when measured on Reflotron compared to serum ALT values measured with a reference method. The authors observed excellent concordance of ALT results when whole blood and plasma were both analyzed using the Reflotron, and similarly when serum was analyzed by the Reflotron and reference method, supporting their conclusion that there was a disagreement between serum and whole blood values.

Further studies will be required to definitively conclude that there is an inherent difference in ALT concentration between venous and capillary blood. We note that the presence of a cover film on DFA paper devices used for testing whole blood was shown in prior experiments during device optimization to slow evaporation that otherwise reduced ALT values measured in whole blood compared to values measured in the same volume of serum with the same ALT concentration. Because use of the cover film for devices used with whole blood completely corrected the effect of evaporation on that sample type and equalized results obtained from whole blood and serum, devices used for fingerstick blood (but not serum) in this study similarly had a cover film. It is possible that the evaporation effect was not completely corrected for fingerstick blood, thus contributing to our findings of lower ALT values in fingerstick vs serum samples, but we find this unlikely. Regardless, the systematic difference we observed between DFA paper test results for fingerstick blood vs serum samples allowed us to calculate a correction factor that can now be applied to fingerstick results to allow them to match serum results obtained from the paper test (which in turn are in close agreement with automated platform results). Therefore, by using this correction factor to adjust the read guide, results of fingerstick testing with the DFA paper test will be calibrated to results of testing serum on the Abaxis Piccolo platform. Further clinical evaluation using the newly adjusted read guide will need to be performed in order to formally validate this correction factor.

This study has further demonstrated the feasibility of remote device reading, in which an image of the device and read guide captured by a simple cell phone camera can be texted to a central location and read there (on a computer screen) by a trained reader. Reads of both 2MP and 8MP camera images were in excellent agreement with the reads of the device in real time (Fig 4). This approach could allow the test to be performed without the need for a reader familiar with device results interpretation to be present on site, and thus opens the door to home-based testing.

In conclusion, we have demonstrated excellent agreement between the DFA paper test and automated platforms for measurement of ALT, and shown that remote reading of the paper device is feasible. Our findings also shed important light on two key aspects of clinical ALT measurement: a) systematic differences in ALT levels by sample type (fingerstick vs serum), and b) variability in ALT measurements provided by different FDA-approved automated platforms. This POC device has significant global potential for monitoring for DILI in patients on potentially hepatotoxic medications, including those for TB and HIV.
